# The Modulation of Ubiquinone, a Lipid Antioxidant, on Neuronal Voltage-Gated Sodium Current

**DOI:** 10.3390/nu14163393

**Published:** 2022-08-18

**Authors:** Te-Yu Hung, Sheng-Nan Wu, Chin-Wei Huang

**Affiliations:** 1 Department of Pediatrics, Chi-Mei Medical Center, Tainan 71004, Taiwan; 2 Department of Physiology, National Cheng Kung University Medical College, Tainan 70101, Taiwan; 3 Institute of Basic Medical Sciences, National Cheng Kung University Medical College, Tainan 70101, Taiwan; 4 Department of Medical Research, China Medical University Hospital, China Medical University, Taichung 40402, Taiwan; 5 Department of Neurology, National Cheng Kung University Hospital, College of Medicine, National Cheng Kung University, Tainan 70101, Taiwan

**Keywords:** coenzyme Q10, ubiquinone, voltage-gated Na^+^ current, persistent Na^+^ current, voltage hysteresis, current kinetics

## Abstract

Ubiquinone, composed of a 1,4-benzoquinone and naturally produced in the body, actively participates in the mitochondrial redox reaction and functions as an endogenous lipid antioxidant, protecting against peroxidation in the pituitary-dependent hormonal system. However, the questions of if and how ubiquinone directly affects neuronal ionic currents remain largely unsettled. We investigated its effects on ionic currents in pituitary neurons (GH3 and MMQ cells) with the aid of patch-clamp technology. Ubiquinone decreased the peak amplitude of the voltage-gated Na^+^ current (*I*_Na_) with a slowing of the inactivation rate. Neither menadione nor superoxide dismutase modified the ubiquinone-induced *I*_Na_ inhibition. In response to an isosceles-triangular ramp pulse, the persistent *I*_Na_ (*I*_Na(P)_) at high- and low- threshold potentials occurred concurrently with a figure-eight hysteresis loop. With ubiquinone, the *I*_Na(P)_ increased with no change in the intersection voltage, and the magnitude of the voltage-dependent hysteresis of the current was enhanced. Ubiquinone was ineffective in modifying the gating of hyperpolarization-activated cation currents. In MMQ lactotrophs, ubiquinone effectively decreased the amplitude of the *I*_Na_ and the current inactivation rate. In sum, the effects of ubiquinone demonstrated herein occur upstream of its effects on mitochondrial redox processes, involved in its modulation of sodium channels and neuronal excitability.

## 1. Introduction

Ubiquinone (coenzyme Q10, ubidecarenone, 2-methyl-5,6-dimethyoxy-1,4-benzoquinone) is a highly lipophilic molecule with a chemical structure similar to that of vitamin K. It is sold as a dietary supplement and is an ingredient in some cosmetics [1,2,3,4,5]. The Q in the term coenzyme Q10 refers to the quinone chemical group and the 10 refers to the number of isoprenyl chemical subunits in its tail (i.e., 10 isoprene units). Ubiquinone belongs to a group of compounds that are characterized by their quinone moieties in addition to the length and composition of their hydrophobic isoprenoid tails.

Ubiquinone, with a quinine derivative and a long isoprenoid tail, has been shown to serve as an intracellular antioxidant in its reduced form, ubiquinol, and to facilitate the production of adenosine trisphosphate in the mitochondria by participating in redox reactions within the electron transport chain [6,7,8]. It has also exhibited benefits for patients with heart failure [4,9,10,11] and various neurological disorders. Ubiquinone is one of the most frequently prescribed agents for treating mitochondrial disorders, such as mitochondrial encephalomyopathy, lactic acidosis, and stroke-like episodes (MELAS) [12]. Moreover, it has been demonstrated to be efficacious and well-tolerated in migraine prophylaxis [13].

There is evidence that pituitary tumors are causally linked to defects in succinate dehydrogenase and deficiencies in the mitochondrial complex by means of which ubiquinone is produced [14]. Changes in the level of ubiquinone have also been associated with hypothalamic–pituitary–adrenal axis dysfunction [15,16]. In addition, ubiquinone and taurine may provide neuroprotection in the hypothalamus and pituitary gland against chlorpromazine-induced neuroendocrine changes [17]. Another study has shown that the coadministration of ubiquinone and lepidium sativum significantly improves the function of the hypothalamic–pituitary–gonadal axis and enhances reproductive parameters [18]. Finally, quinone-derived compounds have proved effective at modifying voltage-gated K^+^ currents in GH_3_ pituitary tumor cells [19]. However, the ionic mechanisms involved in the effects of ubiquinone on pituitary cells remain poorly understood.

There are nine isoforms of voltage-gated Na^+^ (Na_V_) channels (i.e., Na_V_1.1–1.9, or SCN1 A-SCN5 A and SCN8 A-SCN11 A), which are expressed in mammalian excitable tissues, including those in the endocrine system [20,21]. Of note, several inhibitors such as gomisin A (gom A), ranolazine (ran), and KMUP-1 have been found to preferentially block the late component of the voltage-gated Na^+^ current (*I*_Na_) [22,23,24]. Moreover, some Na_V_-channel activators such as pyrethroids and telmisartan have been found in various types of excitable cells [22,25,26,27]. In addition, the *I*_Na_ can be robustly evoked in response to changes in the membrane potential sensed by the channel’s voltage-sensor domain, which is intimately linked to the pore domain [28,29,30,31]. However, at present, few studies provide a comprehensive examination of if and how ubiquinone is capable of interacting directly with Na_V_ channels to perturb the magnitude, gating properties, and hysteretic strength of the *I*_Na_ in excitable cells.

In light of the considerations stated above, in this study, we explore the effect of ubiquinone on different types of ionic currents in GH_3_ pituitary tumor cells: the voltage-gated Na^+^ current (*I*_Na_), the persistent Na^+^ current (*I*_Na(P)_), the *erg*-mediated K^+^ current (*I*_K(erg)_), and the hyperpolarization-activated cation current (*I*_h_). Our findings show that ubiquinone is capable of modifying the amplitude and gating of the *I*_Na_ as well as the voltage-dependent hysteresis of the *I*_Na(P)_, and that it mildly inhibited the amplitude of the *I*_K(erg)_. In addition to being a regulator of mitochondrial biogenesis [6,7,14], the membrane-perturbing properties of ubiquinone demonstrated herein may be shown to contribute, to a certain extent, to its pharmacological and therapeutic effects on pituitary cells appearing in vivo.

## 2. Materials and Methods

### 2.1. Chemicals, Drugs, and Solutions Used in This Work

Ubiquinone (CoQ10, ubidecarenone, 2-methyl-5,6-dimethyoxy-1,4-benzoquinone, 2-[(2E,6E,10E,14E,18E,22E,26E,30E,34E)-3,7,11,15,19,23,27,31,35,39-Decamethyltetraconta-2,6,10,14,18,22,26,30,34,38-decaenyl]-5,6-dimethoxy-3-methylcyclohexa-2,5-diene-1,4-dione, C_59_H_90_O_4_, CAS number: 303-98-0, ≥98% in purity), ivabradine (IVA), menadione (2-methyl-1,4-naphthoquinone), superoxide dismutase, tefluthrin (tef), tetraethylammonium chloride (TEA), and tetrodotoxin (TTX) were acquired from Sigma-Aldrich (Merck Ltd., Tainan, Taiwan). Gomisin A (gom A) was obtained from ChemFaces (Rainbow Biotechnology, Taipei, Taiwan) and ranolazine (ran) from Tocris Cookson (Bristol, UK). To protect the ubiquinone from light degradation [32,33], a stock solution containing this compound was wrapped in aluminum foil. Unless stated otherwise, the culture medium (e.g., F-12 medium), horse or fetal bovine serum, L-glutamine, and trypsin/EDTA were purchased from HyClone^TM^ (Thermo Fisher Scientific, Kaohsiung, Taiwan). Finally, all other chemicals, including aspartic acid, CsCl, CsOH, EGTA, and HEPES, were of laboratory grade and taken from standard sources.

The ionic composition (in mM) of the extracellular solution (normal Tyrode’s solution) used in this study was: NaCl 136.5, KCl 5.4, CaCl_2_ 1.8, MgCl_2_ 0.53, glucose 5.5, and HEPES-NaOH buffer 5.5 (pH 7.4). To measure the *I*_Na_, we bathed cells in Ca^2+^-free Tyrode’s solution, in an attempt to minimize contamination by Ca^2+^-activated K^+^ currents and voltage-gated Ca^2+^ currents [34]. To record the *I*_h_ and the delayed rectifier K^+^ current (*I*_K(DR)_), we backfilled the patch pipette with an internal solution of the following composition (in mM): K-aspartate 130, KCl 20, KH_2_PO_4_ 1, MgCl_2_ 1, Na_2_ATP 3, Na_2_GTP 0.1, EGTA 0.1, and HEPES-KOH 5 (pH 7.2). To measure the *I*_Na_ and the *I*_Na(P)_, the K^+^ ions in the backfilling solution were replaced with Cs^+^ ions, and the pH in the solution was adjusted to pH 7.2 by adding CsOH. To minimize contamination by Cl^-^ currents, the Cl^-^ ions in the internal solution were replaced with aspartate. All solutions were prepared using demineralized water obtained with the Milli-Q water purification system (Merck, Taipei, Taiwan). On the day of the experiments, the medium and solution were filtered using a 0.22 μm pore filter.

### 2.2. Cell Preparations

We acquired GH_3_ pituitary tumor cells from the Bioresources Collection and Research Center (BCRC-60015, http://catalog.bcrc.firdi.org.tw/BcrcContent?bid=60015, Hsinchu, Taiwan, accessed date 8 July 2022) and then grew the cells in a Ham’s F-12 medium supplemented with 15% (by volume) horse serum, 2.5% (by volume) fetal calf serum, and 2 mM of L-glutamine. The MMQ cell line (ATCC CRL-10609, https://www.atcc.org/products/all/CRL-10609.aspx accessed date 8 July 2022), another established pituitary cell line originally derived from a *Rattus norvegicus* prolactinoma, was obtained from the American Type Cell Collection (Manassas, VA, USA) with the help of Genechain (Kaohsiung, Taiwan). The MMQ cells were maintained in an F-12 medium supplemented with 10% (by volume) fetal bovine serum and 1.176 g/L of NaHCO_3_ [35]. The GH_3_ and MMQ cells were grown in a humidified incubator at 37 °C with 5% CO_2_. The viability of these cells was quantified by means of the trypan blue exclusion test. Subcultures were obtained by trypsinization, with 0.025% trypsin solution (HyClone^TM^) containing 0.01% sodium *N*,*N*-diethyldithiocarbamate and EDTA. The experiments were undertaken five or six days after the cells were cultured until they reached 60–80% confluence.

### 2.3. Electrophysiological Measurements

Before each measurement was taken, the GH_3_ or MMQ cells were gingerly dispersed with a 1% trypsin/EDTA solution, and an aliquot of the cell suspension was then placed in a home-made recording chamber mounted on the stage of an inverted DM-IL fluorescence microscope (Leica; Major Instruments, New Taipei City, Taiwan). We immersed the cells at room temperature (20–25 °C) in a HEPES-buffered normal Tyrode’s solution, the composition of which was stated above. To prepare the recording electrode, Kimax-51 borosilicate-glass capillaries with an outer diameter of 1.5 to 1.8 mm (#34500; Kimble; Dogger, New Taipei City, Taiwan) were used, and they were fabricated with a two-step vertical puller (model PP-83; Narishige, Taiwan Instrument, Taipei, Taiwan). We also fire-polished the pipettes’ tips by using a Narishige microforge (Model MF-83). The resistances of the patch electrodes were measured and maintained between 3 and 5 MΩ. Measurements of the ionic currents were made in the whole-cell configuration of the modified patch-clamp technique (i.e., a decremental change in the suction pressure in response to a progressive increase in the seal resistance) with an RK-400 patch-clamp amplifier (Biologic, Claix, France) [34]. As has previously been observed [36], the formation of membrane blebs inside the pipette tip was consistently noticed during the microscopic observation of the giga-seal formation. The junction potentials, which develop at the pipette tip when the composition of the internal solution is different from that of the bath, were nulled before the start of each seal formation, and a junction potential correction was applied to the whole-cell data. The tested compounds were applied through perfusion or were added to the bath to achieve the final concentration indicated.

### 2.4. Potential and Current Recordings

The signals (i.e., current and potential tracings) were displayed on a GW oscilloscope (GOS-622G; Good Will, Taipei, Taiwan) or on a Hantek-6022BC oscilloscope (Qingdao, China). The data were digitally sampled and stored at 10 kHz in an ASUSPRO-BU401LG computer (ASUS, Tainan, Taiwan). With the help of a Digidata 1440A converter (Molecular Devices), data acquisition was carried out using pCLAMP 10.7 software (Molecular Devices). While the measurements were being recorded, the acquired current and potential tracings were also displayed on an MB169B+ monitor (MB169B+; ASUS) with a universal series bus (USB) type-C connection. The laptop computer used was placed on top of an adjustable Cookskin stand (Ningbo, China) for efficient manipulation. A low-pass filter was applied to current signals at 2 kHz with an FL four-pole Bessel filter (Dagan, Minnepolis, MN, USA) to minimize electrical noise. Through digital-to-analog conversion, the pCLAMP-generated voltage profiles, composed of various types of rectangular and ramp pulses, were specially designed and then delivered to determine the current-voltage (*I–V*) relationships and the voltage-dependent hysteresis of the different ionic currents under observation (i.e., *I*_Na_ and *I*_Na(P)_). To ensure digitalization during the recordings, separate sets of experiments were performed using a PowerLab 2/26 acquisition system (AD Instruments; KYS Technology, Tainan, Taiwan). In order to prevent possible light-induced derangements in the ubiquinone molecule [32,33], electrophysiological recordings were made in the absence of illumination.

### 2.5. Data Analyses

To determine the concentration–response relationship for the effect of the ubiquinone on the inhibition of the peak *I*_Na_, cells were bathed in Ca^2+^-free Tyrode’s solution, and the recording pipette was backfilled with a Cs^+^-containing solution. To measure the ion flow of the *I*_Na_, each cell was depolarized from −80 to −10 mV for 40 ms, and the current amplitude was measured at the start of each depolarizing step. The peak amplitudes of the *I*_Na_ (i.e., TTX-sensitive *I*_Na_) in the presence of different concentrations of ubiquinone were compared after the subsequent addition of 1 μM of TTX. The concentration–response curve was described with the modified Hill function, a three-parameter logistic model:

$$\mathrm{Percentage}\;\mathrm{inhibition}\;\left(\%\right)=\frac{{\left[\mathrm{ubiquinone}\;\right]}^{n_{H}}\times E_{\max}}{{\mathrm{IC}}_{50}^{n_{H}}+{\left[\mathrm{ubiquinone}\;\right]}^{n_{H}}}$$

where IC_50_ and n_H_ are defined as the 50% inhibitory concentration (i.e., the concentration producing the response midway between the estimates for the minimum and the maximum) and the Hill coefficient, respectively; ubiquinone represents the concentration of the coenzyme Q10 applied; and E_max_ denotes the maximal inhibition of the peak *I*_Na_ (i.e., the TTX-sensitive current) produced by ubiquinone.

### 2.6. Curve-Fitting Procedures and Statistical Analyses

The least-squares method was used to fit the experimental data to linear and nonlinear (e.g., the single and double exponential function, and the modified Hill function) regression models with the help of either the Excel Solver (Microsoft, Redmond, WA, USA) or the 64-bit OriginPro 2021 (OriginLab; Scientific Formosa, Kaohsiung, Taiwan). The whole-cell voltage-clamp data are expressed as the mean ± SEM with sample sizes (n) denoting the number of GH_3_ and MMQ cells from which the data were acquired, and error bars are plotted as SEM. To assess the assertions with respect to the variability of the mean, the Student’s *t*-test (paired and unpaired samples) or the one-way analysis of variance (ANOVA) test was used, followed by the post-hoc Fisher’s least significant difference test. Values of *p* < 0.05 were considered statistically significant.

## 3. Results

### 3.1. Effects of Ubiquinone on I_Na_ in GH_3_ Pituitary Cells

**Figure 1:**
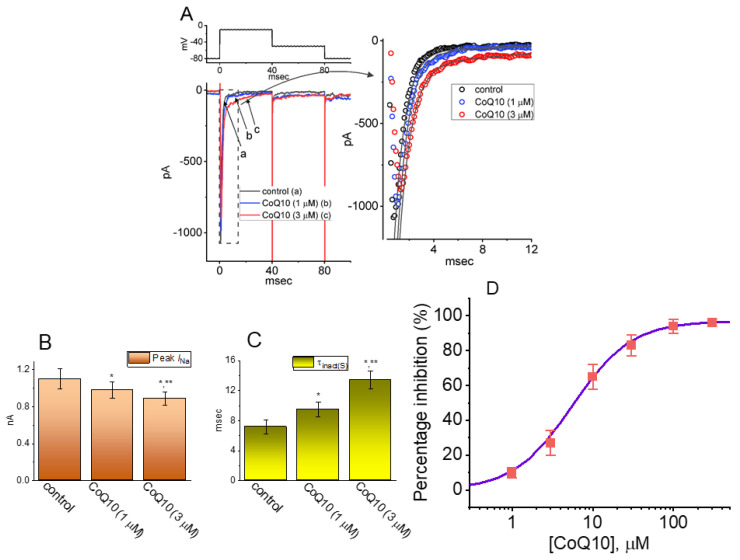

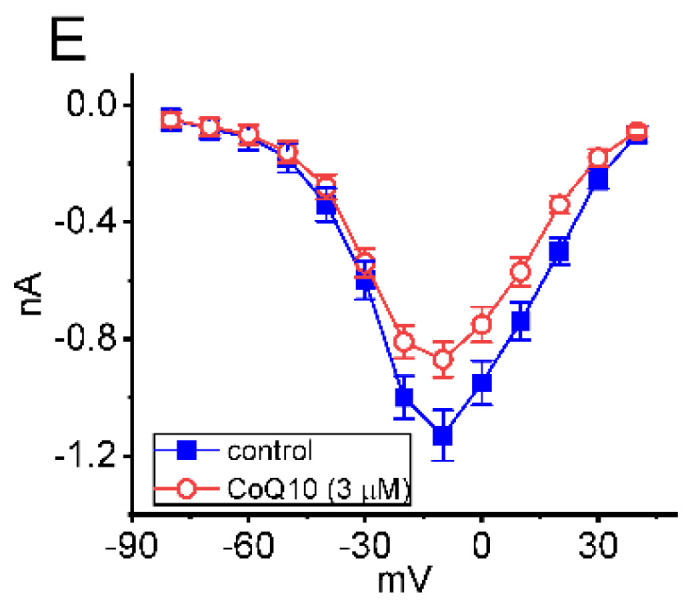
Effects of Ubiquinone (CoQ10) on voltage-gated Na^+^ current (*I*_Na_) measured from GH_3_ pituitary cells. In these experiments, we kept cells immersed in Ca^2+^free Tyrode’s solution containing 10 mM of TEA, and the recording pipette was filled with a Cs^+^-containing solution. The composition of these solutions is detailed in the **Materials and Methods** section. (**A**) Representative *I*_Na_ traces taken (a) during the control period and during cell exposure (b) to 1 μM of CoQ10 or (c) to 3 μM of CoQ10. The right side of panel (**A**) indicates an expanded record taken from the dashed box on the left side, while the upper part shows the voltage-clamp profile used. The smooth gray line denotes the double exponential (i.e., the fast and slow components) fit that has been overlaid on the curve representing the current (open symbols). The summary bar graphs shown in (**B**), orange bars and (**C**), yellow bars illustrate the effects of CoQ10 (1 and 3 μM) on both the amplitude and the slow component of the inactivation time constant (τ_inact(S)_), respectively, of the *I*_Na_ evoked in response to brief depolarizing voltage steps. Both the amplitude (i.e., at the beginning of voltage pulse) and the inactivation time constant (i.e., τ_inact(S)_ of the current with and without the addition of CoQ10) were measured when the GH_3_ cells were depolarized from −80 to −10 mV in a period of 40 ms. Each point represents the mean ± SEM (n = 8). * Significantly different from the control group (*p* < 0.05) and ** significantly different from the addition of CoQ10 (1 μM) alone group (*p* < 0.05). (**D**) The concentration-dependent inhibition of the peak *I*_Na_ produced by CoQ10. Each cell was voltage-clamped at −80 mV, and the current amplitude recorded during cell exposure to different concentrations of CoQ10 was measured at the start of a 40 ms-long depolarizing voltage step to −10 mV. The values of IC_50_, n_H,_ and E_max_ related to the CoQ10-induced inhibition of the peak *I*_Na_ were estimated to be 5.6 μM, 1.2, and 97%, respectively. The continuous sigmoidal curve, over which the data points were overlaid, demonstrates the best fit to the modified equation indicated in the **Materials and Methods** section. (**E**) Steady-state current versus voltage (*I–V*) relationships of peak *I*_Na_ obtained in the absence (■) and presence (○) of 3 μM CoQ10 (mean ± SEM; n = 7 for each point). Peak current amplitude was measured at the start of each depolarizing voltage step.

In the first stage of the experiments, recordings of whole-cell voltage-clamp currents were employed to assess the perturbations caused by ubiquinone on the *I*_Na_ in GH_3_ pituitary cells. To record the *I*_Na_, we suspended cells in Ca^2+^-free Tyrode’s solution and then backfilled the recording pipette with a K^+^-enriched internal solution. When the whole-cell configuration was firmly established, the examined cell was voltage-clamped at −80 mV, and then 40 ms-long depolarizing voltage commands to −10 mV were applied followed by a return to −50 mV in order to evoke the *I*_Na_ [25,37]. Approximately one minute after the cells were exposed to different concentrations of ubiquinone, the *I*_Na_ progressively decreased in response to the abrupt step depolarization, and, concurrently, the inactivation and deactivation time courses of the current became slower (Figure 1). For example, when we depolarized to −10 mV from a holding potential of −80 mV, ubiquinone at a concentration of 3 μM produced a reduction in the peak amplitude (i.e., measured at the start of the depolarizing pulse) of the *I*_Na_ from 1.1 ± 0.11 to 0.91 ± 0.07 nA (n = 8, *p* < 0.05). The presence of ubiquinone at a concentrations of 3 or 10 μM also brought about a concomitant increase in the slow component of the inactivation time constant (τ_inact(S)_) to 9.5 ± 1.01 ms (n = 8, *p* < 0.05) or 13.4 ± 1.22 ms (n = 8, *p* < 0.05), respectively, from a control value of 7.18 ± 0.91 ms (n = 8). After the compound was washed out, the amplitude of the current returned to 1.05 ± 0.09 nA (n = 8, *p* < 0.05). Figure 1B,C show the summary bar graphs demonstrating the effects of ubiquinone (1 and 3 μM) on the peak amplitude and the τ_inact(S)_ of the current, respectively. 

We further examined the relationship between the concentration of ubiquinone and the percentage of inhibition of the peak *I*_Na_. Figure 1D illustrates that the peak amplitude of the *I*_Na_ activated during a short step depolarization from −80 to −10 mV was variously depressed in the presence of ubiquinone depending on its concentration, with an estimated IC_50_ of 5.6 μM, and ubiquinone at a concentration of 100 μM decreased *I*_Na_ by approximately 97%. Figure 1E illustrates the overall *I–V* relationships of peak *I*_Na_ with or without cell exposure to 3 μM ubiquinone, reflecting that ubiquinone did not alter the *I–V* curve of the current. Therefore, the results indicate, first, that, under our experimental conditions, the *I*_Na_ remained functionally active in the GH_3_ cells [21,23,26,27,34,38], and, second, that the presence of ubiquinone may play a modulatory role in relation to the magnitude and the inactivation time course of the *I*_Na_ produced by the abrupt depolarization of the cell membrane.

### 3.2. Comparison between the Effects of Ubiquinone Alone and Those of Ubiquinone plus Menadione, Ubiquinone plus Superoxide Dismutase, and Ubiquinone plus Tefluthrin (Tef) on the Peak I_Na_

**Figure 2:**
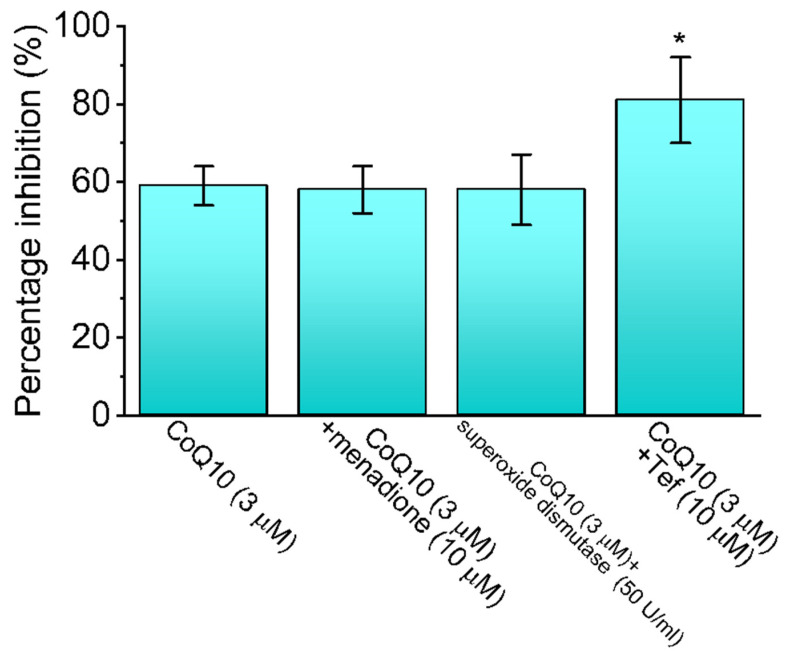
Summary bar graph showing effects (i.e., percentage inhibition) of ubiquinone (CoQ10), CoQ10 plus menadione, CoQ10 plus superoxide dismutase, and coQ10 plus tefluthrin (tef) (mean ± SEM; n = 8 for each bar). The results reflect that CoQ10-mediated inhibition of *I*_Na_ is unlinked to the level of reactive oxygen species. Peak amplitudes of *I*_Na_ with or without different tested compounds were compared. * Significantly different from CoQ10 (3 μM) alone group (*p* < 0.05).

Oxidative stress readily affects the integrity of the cell membrane, and redox reagents may react with essential cysteine groups located on the channel proteins [16,39,40]. Therefore, the effects of ubiquinone alone, as well as those of ubiquinone plus menadione, ubiquinone plus superoxide dismutase, and ubiquinone plus tef on the peak *I*_Na_ recorded from GH_3_ cells were first examined and then compared. In terms of their individual characteristics, menadione is an oxidizing agent [41], superoxide dismutase is capable of reducing the damage caused by lipid peroxidation to surface membranes [42], and tef shows potential as an effective activator of the *I*_Na_ [26]. As shown in Figure 2, in the continued presence of 3 μM of ubiquinone, the subsequent addition of neither menadione (10 μM) nor superoxide dismutase (500 U/mL) altered the ubiquinone-mediated inhibition of the peak *I*_Na_; however, the addition of tef (10 μM) was effective at reversing the decrease in the peak *I*_Na_. tef (10 μM) alone increased peak *I*_Na_ from 1012 ± 25 to 1623 ± 29 pA (n = 7, *p* < 0.05). These results suggest that the peak *I*_Na_ in GH_3_ cells was sensitive to the inhibitory effect of ubiquinone, but that its magnitude remained unaffected after the subsequent addition of menadione or superoxide dismutase. However, tef proved effective at counteracting ubiquinone’s inhibition of the peak *I*_Na_.

### 3.3. Effects of Ubiquinone on the Voltage-Dependent Hysteresis of the Persistent I_Na_ (I_Na(P)_) in GH_3_ Cells

**Figure 3:**
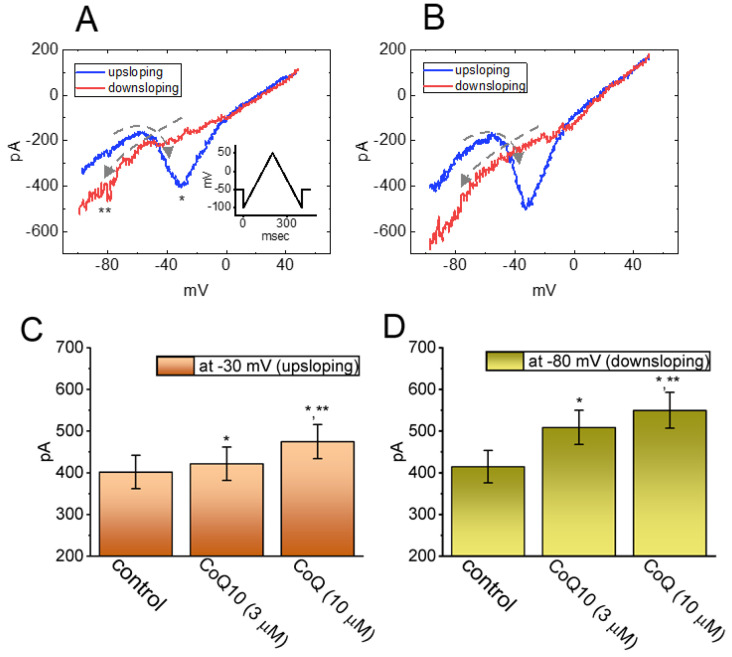
Effects of ubiquinone (CoQ10) on the voltage-dependent hysteresis of the persistent *I*_Na_ (*I*_Na(P)_) in GH_3_ cells. The cells were bathed in Ca^2+^-free Tyrode’s solution, and the recording pipette was filled with a Cs^+^-containing solution. When the whole-cell configuration was established, the examined cell was voltage-clamped at −50 mV, and then an upright isosceles-triangular ramp pulse was applied covering the voltage range from −100 to +50 mV for a duration of 200 ms (or a ramp speed of ±0.75 V/s). Panels (**A**,**B**) present representative current traces obtained during the control period and during cell exposure to 3 μM CoQ10, respectively. The inset in (**A**) shows the voltage-clamp protocol applied. The dashed lines in (**A**,**B**) indicate the direction of the trajectory of the *I*_Na(P)_ following the passage of time, indicating a figure-eight configuration of the current. * and ** denote the high-threshold *I*_Na(P)_ and the low-threshold *I*_Na(P)_, respectively, elicited by the ascending (upsloping, in blue color) and descending (downsloping, in red color) limb of a 400 ms-long isosceles-triangular ramp pulse. The summary bar graphs in (**C**,**D**) depict the effects of CoQ10 (3 and 10 μM) on the amplitude of the high-threshold *I*_Na(P)_ and the low-threshold *I*_Na(P)_, respectively, in the GH_3_ cells (mean ± SEM; n = 8 for each bar). The amplitudes of the high-threshold *I*_Na(P)_ and the low-threshold *I*_Na(P)_ activated by a 200 ms-long isosceles-triangular ramp pulse were measured at −30 and −80 mV, respectively. * Significantly different from the control group (*p* < 0.05) and ** significantly different from the addition of CoQ10 (3 μM) alone group (*p* < 0.05).

The voltage-dependent hysteresis of various ionic currents has recently been shown to have a significant impact on electrically excitable cells [30,38]. Therefore, we further explored whether the presence of ubiquinone resulted in perturbing the voltage-dependent hysteresis inherently existing in the *I*_Na(P)_ recorded from GH_3_ cells. In this set of whole-cell current recordings, we digitally generated a long-lasting isosceles-triangular ramp pulse over the voltage range of −100 to +50 mV for a duration of 200 ms (i.e., ±0.75 V/s). When the whole-cell configuration was firmly established, we gingerly delivered this pulse to the cells through digital-to-analog conversion. As seen in Figure 3, of particular interest is the fact that the trajectories of the *I*_Na(P)_ responding to the ramp pulse ascending from −100 to +50 mV and to the ramp pulse descending from +50 to −100 mV as a function of time began to emerge and become distinct from one another [38]. These trajectories formed a figure-eight loop in the instantaneous *I–V* relationship of the current with the intersection point of around −45 mV. As indicated by the dashed lines in Figure 3A,B, a hysteresis loop in the counterclockwise direction was noticed above the intersection point, whereas below this point it became clockwise. As an isosceles-triangular ramp pulse with a duration of 200 ms (or a ramp speed of ±0.75 V/s) was being applied to the cells, the amplitude of the peak *I*_Na(P)_ during the control period was measured at −30 mV (i.e., a high-threshold *I*_Na(P)_) after being activated during the ascending phase of the triangular ramp pulse. In contrast, the amplitude of the peak *I*_Na(P)_ was measured at −80 mV (i.e., a low-threshold *I*_Na(P)_) during the descending phase of the triangular ramp pulse. One minute after the cells were continually exposed to ubiquinone (3 μM), the amplitudes of both the high- and low-threshold *I*_Na(P)_ were found to rise. Figure 3C,D present the summary bar graphs of the stimulatory effects of ubiquinone (3 and 10 μM) on the high-threshold *I*_Na(P)_ and the low-threshold *I*_Na(P)_, respectively, which were activated by an upright isosceles-triangular ramp pulse. However, no change in the level of the intersection point was recorded. It is conceivable, therefore, that the addition of ubiquinone triggered an increase in the strength of voltage-dependent hysteresis of the instantaneous *I–V* relationship of the *I*_Na(P)_ in response to the isosceles-triangular ramp voltage applied to the GH_3_ cells.

### 3.4. Mild Inhibition of Erg-Mediated K^+^ Current (I_K(erg)_) Produced by Ubiquinone in GH_3_ Cells

**Figure 4:**
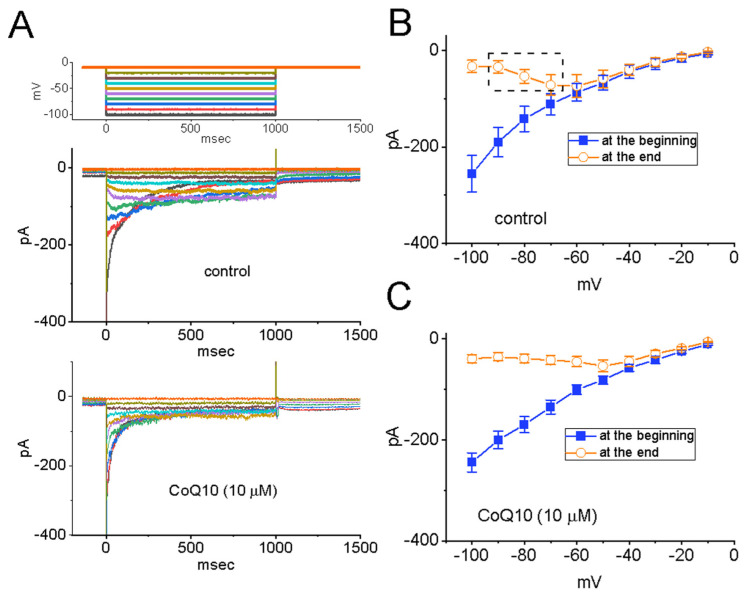
Mild inhibitory effect of ubiquinone (CoQ10) on the *erg*-mediated K^+^ current (*I*_K(erg)_) in GH_3_ cells. In these experiments, the cells were immersed in a high-K^+^ (around 140 mM of K^+^), Ca^2+^-free solution, and the recording pipette was filled with a K^+^-containing internal solution. (**A**) Representative current traces obtained with (their lower part) and without (their upper part) the addition of 10 μM of CoQ10. The uppermost part denotes the voltage-clamp protocol applied to the examined cells. Of note, the potential traces shown in different colors correspond to the current traces activated in response to each hyperpolarizing voltage. (**B**,**C**) show the *I–V* relationships of the peak (■) and late (○) deactivating *I*_K(erg)_ obtained in the control period and during cell exposure to 10 μM of CoQ10, respectively. The current amplitude was recorded at the beginning (■) and the end (○) of each 1 s-long hyperpolarizing voltage pulse from a holding potential of −10 mV. Each point is the mean ± SEM (n = 8, for each point). The dashed box in (**B**) indicates the voltage-dependent inactivation of the *I*_K(erg)_ present in these cells, according to the steady-state *I–V* relationship of sustained *I*_K(erg)_.

A previous report demonstrated the effectiveness of probucol, an anti-oxidative and lipid-lowering agent, at inhibiting the amplitude of the *I*_K(erg)_ [43]. Therefore, we proceeded to examine the possible perturbations caused by ubiquinone on the amplitude of the *I*_K(erg)_ measured from GH_3_ cells. To ensure the presence of the *I*_K(erg)_, we kept the cells bathed in a high-K^+^, Ca^2+^-free solution, and the pipette was filled with a K^+^-containing internal solution. As shown in Figure 4, the deactivation of the *I*_K(erg)_ was robustly activated in response to various levels of membrane potentials starting from a holding potential of −10 mV [43,44]. The effect of ubiquinone on the steady-state *I–V* relationships of the *I*_K(erg)_ in GH_3_ cells was determined. Figure 4A–C show the relationship between the current amplitudes at the start and at the end of the *I*_K(erg)_, which was activated by 1 s-long hyperpolarizing steps starting from a holding potential of −10 mV, and was recorded both with and without the addition of ubiquinone (10 μM). Interestingly, the presence of ubiquinone at a concentration of 10 μM triggered a considerable reduction in the peak or sustained component of the deactivating *I*_K(erg)_ which was responding to step hyperpolarization. For example, cell exposure to 10 μM of ubiquinone appreciably decreased the slope of the fitted line for the whole-cell conductance of *I*_K(erg)_ from 4.8 ± 0.9 to 3.6 ± 0.7 nS (n = 7, *p* < 0.05) at voltages between −100 and −70 mV. Meanwhile, the magnitude of the inactivating current was attenuated in the presence of ubiquinone. These experimental observations indicate that ubiquinone at a concentration of 10 μM is capable of mildly depressing the amplitude of the *I*_K(erg)_ present in GH_3_ cells.

### 3.5. Inability of Ubiquinone to Modify the Amplitude and Gating of Hyperpolarization-Activated Cation Current (I_h_) in GH_3_ Cells

**Figure 5:**
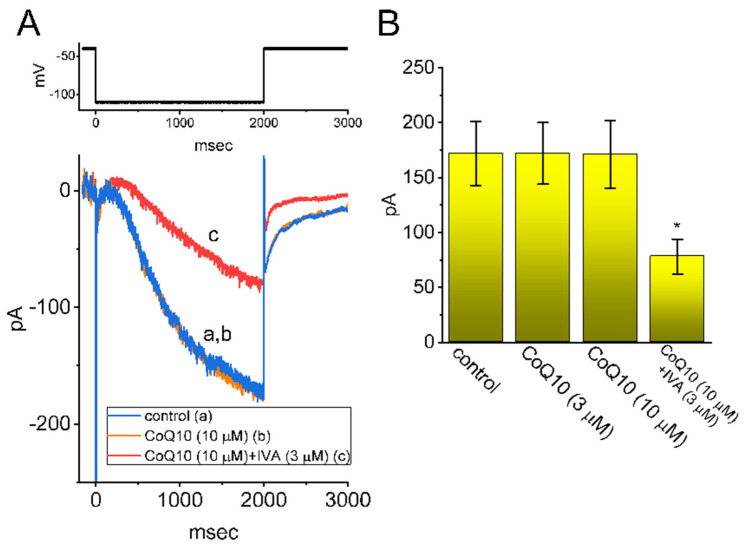
Effects of ubiquinone (CoQ10) on the hyperpolarization-activated cation current (*I*_h_) in GH_3_ cells. The introduction of CoQ10 was ineffective at perturbing the amplitude and gating of the hyperpolarization-activated cation current (*I*_h_) in GH_3_ cells. This set of experiments was conducted in cells which were bathed in Ca^2+^-free Tyrode’s solution containing 1 μM of TTX, and the pipette was filled with a K^+^-containing solution. (**A**) Representative current traces obtained (a) during the control period and during cell exposure (b) to 10 μM of CoQ10 and (c) to 10 μM of CoQ10 plus 3 μM of IVA. The voltage-clamp protocol applied is illustrated in the upper part. (**B**) A summary bar graph showing the effects of CoQW10 and CoQ10 plus IVA on the amplitude of the *I*_h_ (mean ± SEM; n = 8, for each bar). The current amplitude was measured at the endpoint of the hyperpolarizing step pulse from −40 to −110 mV for a duration of 2 s. * Significantly different from the control group (*p* < 0.05).

In another stage of our experiments, we investigated whether the *I*_h_ inherently present in GH_3_ cells was subject to any modification caused by the introduction of ubiquinone. The cells were bathed in Ca^2+^-free Tyrode’s solution, and the pipette was filled with a K^+^-containing solution when the recordings were taken. As shown in Figure 5A, after the whole-cell configuration was established, the examined cell was hyperpolarized for 2 s to −110 mV from a holding potential of −10 mV, and the slowly activating *I*_h_ was robustly activated [45,46]. The addition of ubiquinone at concentrations of 3 and 10 μM failed to modify the amplitude and activation time course of the *I*_h_ evoked in response to the long-lasting membrane hyperpolarization. However, in the continued presence of ubiquinone (10 μM), the further addition of ivabradine (3 μM) effectively decreased the amplitude of the *I*_h_ and also slowed down the activation rate of the current. This result may be explained by the previous finding that ivabradine is capable of inhibiting the *I*_h_ [46] (Figure 5B). It is clear from the current observations that, as compared with the *I*_Na_ and the *I*_Na(P)_, the *I*_h_ present in GH_3_ cells is relatively resistant to modification by the addition of ubiquinone.

### 3.6. Inhibitory Effects of Ubiquinone on I_Na_ in MMQ Pituitary Cells

**Figure 6:**
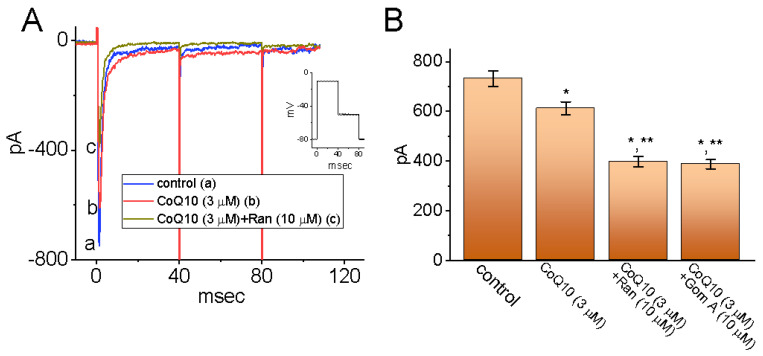
Effects of ubiquinone (CoQ10) on the *I*_Na_ in MMQ pituitary cells. The whole-cell experiments were conducted with MMQ cells, which were kept in Ca^2+^-free Tyrode’s solution, and we backfilled the pipette with a Cs^+^-containing solution. (**A**) Representative current traces obtained (a) during the control period and during cell exposure (b) to 3 μM of CoQ10 and (c) to 3 μM of CoQ10 plus 10 μM of ran. The inset demonstrates the voltage-clamp protocol applied to the cell. (**B**) A summary bar graph showing the effects of CoQ10, CoQ10 plus ran, and CoQ10 plus gom A (mean ± SEM; n = 8). The current amplitude was measured at the beginning of the depolarizing pulse from −80 to −10 mV. * Significantly different from the control group (*p* < 0.05), and ** significantly different from the addition of CoQ10 (3 μM) alone group (*p* < 0.05).

It is possible that the biophysical properties of the *I*_Na_ in GH_3_ cells that have been discussed are different from those of such currents in other types of lactotrophs. Thus, the effects of ubiquinone on the *I*_Na_ in another type of pituitary cell, MMQ cells, was measured in a separate set of recordings. In this set of experiments, we kept the cells immersed in Ca^2+^-free Tyrode’s solution containing 10 mM of TEA, and the recording pipette was filled with a Cs^+^-containing solution. Similar to the findings for GH_3_ cells, an *I*_Na_ with rapid activation and inactivation time courses appeared to be generated in the MMQ cells in response to short depolarizing steps. Also of note is the fact that, when the MMQ cells were continually exposed to ubiquinone (3 μM), the peak amplitude of the *I*_Na_ evoked by an abrupt depolarization from −80 to −10 mV was significantly decreased from 732 ± 32 to 612 ± 26 pA (n = 8, *p* < 0.05) (Figure 6A,B). Furthermore, the addition of ran (10 μM) and gom A (10 μM), while maintaining a concentration of 3 μM of ubiquinone, was effective at decreasing the peak *I*_Na_ even further (Figure 6B), as demonstrated by the significant reduction in the peak *I*_Na_ to 397 ± 21 pA (n = 8, *p* < 0.05) in the case of ran and to 387 ± 19 pA (n = 8, *p* < 0.05) in the case of gom A. Ran and gom A have been shown to block the *I*_Na_ effectively [22,24,47,48]. Therefore, it appears that GH_3_ and MMQ pituitary cells have in common a sensitivity to the inhibitory effects of ubiquinone on the amplitude and gating of the *I*_Na_. 

## 4. Discussion

The present study provides evidence that the exposure of GH_3_ and MMQ cells to ubiquinone produces an inhibitory effect on the depolarization-evoked peak *I*_Na_ concurrently with a slowing in current inactivation. When the time course of the current inactivation, evoked in response to the depolarizing voltage steps, was fitted to a double exponential function, the slow component of the inactivation time constant (τ_inact(S)_) of the *I*_Na_ noticeably increased in the presence of ubiquinone. Therefore, these results led us to conclude that the presence of ubiquinone is capable of modifying the amplitude and gating of the *I*_Na_ in GH_3_ cells. 

It should be stressed, based on the current investigations, that the nonlinear voltage-dependent hysteresis of the *I*_Na(P)_ during the control period and during cell exposure to ubiquinone was observed during the upright isosceles-triangular ramp voltage command (Figure 3). More specifically, as the cells were exposed to ubiquinone, the peak *I*_Na(P)_ activated on the forward (upsloping) limb of the triangular ramp pulse increased, particularly at the level of −30 mV, whereas the *I*_Na(P)_ on the backward (downsloping) limb increased at −80 mV. Consequently, in the presence of ubiquinone, enhanced measurements were recorded of a figure-eight configuration of the hysteresis loop of the *I*_Na(P)_ elicited in response to the triangular ramp pulse (Figure 3), despite the effectiveness of ubiquinone at decreasing the peak *I*_Na_ activated by short depolarizing pulses. In other words, when the isosceles-triangular ramp pulse was applied to the cells, two types of *I*_Na(P)_ appeared to emerge: a low-threshold *I*_Na(P)_ (i.e., activated at a range of voltages near the resting potential) and a high-threshold *I*_Na(P)_ (i.e., activated at a range of voltages near the maximally activated *I*_Na_). This was clearly observed during cell exposure to ubiquinone. However, no discernible change in the voltage level at the intersection point of the hysteresis loop was detected during cell exposure to ubiquinone. The low-threshold *I*_Na(P)_ was noticeably activated on the descending (downsloping) limb of the triangular ramp pulse, whereas the activation of the high-threshold *I*_Na(P)_ occurred on the ascending (upsloping) limb. As the speed of the ramp pulse was decreased, the area of the hysteresis loop became progressively smaller. Therefore, the finding that the triangular pulse-induced *I*_Na(P)_ underwent perturbations in the voltage-dependent hysteresis inherently occurring in GH_3_ cells is consistent with the results of previous studies [30,31,38].

In the current study, while the GH_3_ cells were exposed to ubiquinone, the voltage-dependent movement of the S4 segment in the Na_V_ channels was perturbed; consequently, the coupling of the pore domain and the voltage-sensor domain was enhanced [28,29,30]. The energetic coupling of the voltage sensor during channel activation is assumed to occur in a conformationally flexible region of the protein. It therefore seems reasonable to suggest that an *I*_Na(P)_ evoked during cell exposure to ubiquinone possesses an intrinsic and dynamic “memory” of past events. This can be explained on the basis of a modal shift that occurs with respect to the voltage sensitivity of the gating charge movement, which relies heavily on the previous state (or conformation) of the Na_V_ channel [28,29,30,38]. 

The ubiquinone molecule is known to have amphipathic (biphasic) properties owing to the hydrophilic benzoquinone ring and the lipophilic poly-isoprenoid side-chain [49]. It has been previously demonstrated that the headgroup carbonyl of the ubiquinone molecule tends to attach itself to the polar bilayer of the surface membrane and, concurrently, its chain tends to settle into the hydrocarbon bilayer interior [49,50]. It is likely that the binding speed of the ubiquinone molecule and its affinity for a particular state determine the kinetic behavior of the plasmalemmal *I*_Na_ during cell exposure to ubiquinone. Regardless of the precise details of the mechanism underlying the effects of ubiquinone, the increased hysteretic strength of the *I*_Na(P)_ caused by ubiquinone is expected to affect excitable cells and the rhythmic firing of action potentials. Moreover, it remains to be determined if the effects of ubiquinone on the amplitude and inactivation time course of peak *I*_Na_ are distinct and unrelated. 

Previous reports have demonstrated the ability of ubiquinone to decrease the heart rate [1,4,9]. Ivabradine, known to block the *I*_h_, is a bradycardic agent [46]. The fact that ubiquinone was ineffective at modifying the amplitude and gating of the *I*_h_ in the GH_3_ cells suggests that ubiquinone-induced bradycardia might not be strongly connected with any modifications in the magnitude of the *I*_h_ inherently evoked in sinoatrial cells [51,52]. 

The stimulus–secretion coupling occurring in neuroendocrine and endocrine cells is intimately linked to Ca^2+^ signaling [21]. However, the relationship between Ca^2+^ signaling and the amplitude of action potentials is not simple. For example, spiking of a smaller amplitude but of a longer duration may be associated with stronger Ca^2+^ transients in different types of endocrine cells. In addition, hormone secretion by pituitary lactotrophs (e.g., GH_3_ and MMQ cells) may occur in a Ca^2+^-regulated manner largely through constitutive exocytosis [21]. Therefore, our findings suggest that changes in ion-channel activity caused by ubiquinone-induced perturbations may play a role in changes in the release of growth hormone and prolactin in in vivo pituitary cells [14,17,53]. However, the extent to which the effect of ubiquinone on ion-channel activity is linked to its side effects (e.g., diarrhea and nausea) remains to be further studied. Moreover, since the *I*_Na_ intrinsically in GH_3_ or MMQ cells could be different from those in native (central and/or peripheral) neurons, effects of ubiquinone on ionic currents in different types of neurons still needs to be further evaluated.

The reference levels previously reported for the concentration of ubiquinone in serum range between 0.7 and 1.1 μM [4]. The IC_50_ value of the ubiquinone-mediated inhibition of the peak *I*_Na_ in the GH_3_ cells was 5.6 μM. It should be noted that the effects of ubiquinone on the membrane ionic currents demonstrated herein were rapid in onset and of pharmacological and therapeutic relevance [1,4,5,9,11,54,55,56,57], although ubiquinone is recognized as a mobile electron carrier with a faster diffusion rate than that of bulkier protein complexes. Moreover, with the continued presence of ubiquinone, the addition of menadione and superoxide dismutase was ineffective at reversing the ubiquinone-induced inhibition of the peak *I*_Na_ in the GH_3_ cells, whereas the subsequent addition of tefluthrin attenuated this inhibitory effect. Such perturbations of ionic currents (e.g., *I*_Na_ and *I*_K(erg)_) tend to occur upstream of the ameliorating effects that ubiquinone has on mitochondrial derangements and redox dysfunction [4,5,6,9,11,53,55,56,57].

In terms of its value to pharmacological and therapeutic interventions, ubiquinone has shown a seizure-attenuating effect in animal models of epilepsy, which is assumed to occur by means of the modulation of oxidative stress. The subchronic administration of ubiquinone may attenuate seizures induced by pentylenetetrazole and by electroshock, probably by means of the induction of nitric oxide synthase [58]. Ubiquinone treatment may also attenuate spontaneous recurrent seizures and inhibit hippocampal neuronal loss and aberrant mossy fiber sprouting in the model of kainite-induced temporal lobe epilepsy, which is related in part to the mitigation of oxidative stress by ubiquinone [59]. Similarly, ubiquinone has also proven safe and effective as an adjuvant to phenytoin in treating epilepsy, both for ameliorating seizure severity and for protecting against seizure-induced oxidative damage [60]. As a possible extension of these beneficial effects, the direct neuronal sodium channel-modulating effect of ubiquinone shown in our study is a candidate for the underlying mechanism by means of which ubiquinone is able to alter neuronal hyperexcitability and attenuate seizure-like disorders. We have previously reported the effectiveness of add-on multi-vitamins for treating patients with intractable focal epilepsy [61], and we have also found that the level of ubiquinone in serum correlated with seizure frequency [62]. Therefore, the possibility that ubiquinone has a distinct effect on the reduction of seizures deserves further clinical investigation. 

Ubiquinone shows therapeutic potential for ameliorating glutamate excitotoxicity [63] and as an inhibitor of the release of glutamate from cortical synaptosomes by means of the suppression of the presynaptic voltage-dependent calcium influx [64]. These effects are parallel to the phenomena described in the current study of ubiquinone attenuating neuronal sodium channel activity and modulating neuronal excitability. The mediating effects of ubiquinone on membrane ionic currents, its modulating effects on neuronal excitability, and the neuroprotection that it provides are all possible avenues for future experimentation and clinical applications. 

## 5. Conclusions

The mediating effects of ubiquinone on membrane ionic currents, its modulating effects on neuronal excitability, and the neuroprotection that it provides are all possible avenues for future experimentation and clinical applications. 

## Data Availability

Data are available upon request from correspondence authors.
